# Combination of the Focus-Forming Assay and Digital Automated Imaging Analysis for the Detection of Dengue and Zika Viral Loads in Cultures and Acute Disease

**DOI:** 10.1155/2022/2177183

**Published:** 2022-07-19

**Authors:** Sara Bolívar-Marin, Irene Bosch, Carlos F. Narváez

**Affiliations:** ^1^División de Inmunología, Programa de Medicina, Facultad de Salud, Universidad Surcolombiana, Neiva 410001, Huila, Colombia; ^2^Institute for Medical Engineering and Science, Massachusetts Institute of Technology (MIT), Cambridge, MA 02139, USA

## Abstract

Optimized methods for the detection of *flavivirus* infections in hyperendemic areas are still needed, especially for working with patient serum as a starting material. The focus-forming assay (FFA) reveals critical aspects of virus-host interactions, as it is a quantitative assay to determine viral loads. Automated image analysis provides evaluations of relative amounts of intracellular viral protein at the single-cell level. Here, we developed an optimized FFA for the detection of infectious Zika virus (ZIKV) and dengue virus (DENV) viral particles in cell cultures and clinical serum samples, respectively. Vero-76 cells were infected with DENV-2 (16681) or ZIKV (PRVA BC59). Using a panel of anti-DENV and anti-ZIKV NS1-specific monoclonal antibodies (mAbs), the primary mAbs, concentration, and the optimal time of infection were determined. To determine whether intracellular accumulation of NS1 improved the efficiency of the FFA, brefeldin A was added to the cultures. Focus formation was identified by conventional optical microscopy combined with CellProfiler™ automated image analysis software. The FFA was used with spike assays for ZIKV and clinical specimens from natural infection by DENV-1 and DENV-2. mAb 7744-644 for ZIKV and mAb 724-323 for DENV used at a concentration of 1 *μ*g/ml and a time of 24 hours postinfection produced the best detection of foci when combining conventional counting and automated digital analysis. Brefeldin A did not improve the assessment of FFUs or their digitally assessed intensity at single-cell level. The FFA showed 95% ZIKV recovery and achieved the detection of circulating DENV-1 and DENV-2 in the plasma of acutely ill patients. The combination of the two techniques optimized the FFA, allowing the study of DENV and ZIKV in culture supernatants and clinical specimens from natural infection in hyperendemic areas.

## 1. Introduction

Dengue virus (DENV) and Zika virus (ZIKV) are emerging and re-emerging pathogens, especially in the Americas and the Caribbean region. DENV is responsible for between 284 and 528 million infections and 25,000 fatal cases each year [1]. During the last decade, the number of cases of DENV infection has shown an ascending pattern, with the historically highest number of cases recorded in the region in 2019-2020 [2]. Colombia is one of the countries in which a high number of infections regularly occur, which causes a serious public health problem; therefore, the region tends to devote effort to studying and understanding the virus. Recently, another *flavivirus* arrived on the scene with greater force. In 2015-2016, the ZIKV epidemic occurred in the Americas, causing 170,000 confirmed cases [3]. Prenatal infection by ZIKV is associated with severe congenital consequences such as microcephaly, calcification of the brain parenchyma, ventriculomegaly, and atrophy of the cerebral cortex, among others [4], and neurological disorders such as Guillain-Barré syndrome and encephalitis can present when infection occurs postnatally [5].

Structurally, DENV and ZIKV have high homology between their viral genomes and proteins, reaching close to 60% [6]. They are single-stranded, positive-stranded RNA viruses. The genomes encode three structural proteins, the capsid (C), premembrane (prM), and envelope (E), and seven nonstructural (NS) proteins, NS1, NS2A, NS2B, NS3, NS4A, NS4B, and NS5 [7]. Nonstructural protein 1 (NS1) is a viral protein highly secreted during infection that participates in viral replication, immune regulation, and the pathophysiology of infection, as DENV NS1 has been shown to increase vascular permeability by altering glycocalyx characteristics in human endothelial cells [8–10]. After infection, intracellular NS1 can be detected by cytometry or fluorescence microscopy methods from 6 hours postinfection (hpi) [11]. It has recently been shown that the secretion of NS1 from vertebrate cells, unlike that from mosquito cells, occurs via the trans-Golgi, since the use of protein secretion inhibitors that function via the Golgi pathway, such as brefeldin A (BFA), decreases the secretion of this viral protein from infected cells [12, 13]. Therefore, in theory, the inhibition of transport through the Golgi apparatus by this drug could increase the amount of NS1 protein by favoring its intracellular accumulation, which would facilitate its detection by various methods.

The focus-forming assay (FFA) is a conventional virological method used for the quantification of infectious viral particles [13–15] and viral loads in clinical and experimental samples. FFAs are also widely used in evaluations of the effectiveness of neutralizing antibodies (e.g., for immune responses induced by vaccination against viral agents and in the search for correlates of protection [16]). The principle of the FFA consists of the detection of intracellular viral proteins expressed by infected cells. Specific conjugated antibodies allow the identification of single foci and the subsequent calculation of the viral titer [17, 18]. In the study of flaviviruses, FFAs have also been useful for the quantification of the number of viral particles, the validation of RT-qPCR tests, the measurement of the antiviral effects of potential antiviral drugs, and postvaccination or natural infection neutralizing antibody titer detection assays, among others [9, 12, 19–25]. Traditionally, FFA reading and interpretation are based on conventional optical microscopy, as this technique is most often used for the enumeration of focus-forming units (FFU). However, since the last decade, the use of digital analysis tools for FFAs has significantly increased the precision and number of variables provided compared to those obtained by the use of conventional optical microscopy alone [26, 27]. For example, in hepatitis C virus infection, it was found that the combination of automated imaging software with conventional light microscopy increased the efficiency of FFAs and achieved a method for improved virion quantification [26], avoiding subjective counts produced by laboratory personnel, especially when the relative amount of intracellular protein targeted by the stain is low.

We present a strategy using light microscopy for event enumeration combined with automated digital image processing and analysis. We developed an FFA by identifying variables such as the ideal antibody and its concentration, the optimal infection time, and the differential effects of Golgi protein transport inhibition between DENV and ZIKV infection. The assay also proved useful for the identification of DENV-1, DENV-2, and ZIKV in naturally infected clinical samples and spike assays.

## 2. Materials and Methods

### 2.1. Viruses and Cells

Vero-76 cells (ATCC® CRL1587™) were cultured in flat-bottomed 96-well plates (Costar, ref: 3595, USA) in sterile DMEM (Gibco, ref: 11885-084, USA), supplemented with 5% FBS (Gibco, ref: 16000-044), 100 U/ml penicillin, 100 *μ*g/ml streptomycin, 2 mM L-glutamine (Gibco, ref: 10378-016, USA) at 37°C, and 5% CO_2_ until 80–90% monolayer confluence was obtained for *in vitro* infection in the FFA. The viruses were previously titrated in two laboratories by a conventional FFU assay. The viral titers of the DENV-2 and ZIKV stocks used were 5.9 × 10^6^ and 3.5 × 10^6^ FFU/ml, respectively. The cells were washed with sterile 1X PBS (Gibco, ref: 14190-144, USA) and infected by 10-fold serial dilutions, with 100 *μ*L added per well. The cells were incubated at 37°C and 5% CO_2_ for the indicated time for each condition. Each dilution was performed at least in triplicate for all experiments. A negative control (mock) was also always introduced. The mock corresponded to the Vero-76 cell culture supernatant without the virus.

To carry out the FFA, previously described tests conducted in studies of *rotavirus* and *flavivirus* [14, 27] were used as references.

At different times after infection, the cells were fixed and permeabilized for 30 min with absolute methanol (Merck, ref: 106009, Germany) precooled to −70°C and added at a volume of 150 *μ*L per well. Subsequently, the methanol was removed, and the cells were washed twice with PBS 1X (Gibco, ref: 21600-069, USA) at 70 *μ*L/well. To select the mAb and its ideal concentration, primary anti-NS1 ZIKV-specific mAbs (mAab 7744-644 or mAb 7746-50-130) or NS1 pan-DENV (mAb 724-323 or mAb 271), which were both previously characterized [28], were added as appropriate to PBS 1X-BSA 1% (Sigma-Aldrich, ref: A7906-500G, USA) for dilution to different concentrations. Then, 100 *μ*l of the dilutions was added per well, and the plates were incubated at room temperature for 1 h. At the end of the designated time, the antibodies were removed, and the cells were washed twice with 1X 150 *μ*L PBS per well. Biotin-coupled goat anti-mouse secondary antibody (KPL) diluted 1/2,000 in PBS-BSA 1% was subsequently added at 100 *μ*l per well, and the plates were incubated at room temperature for 1 h. Afterward, the antibody was removed, and the cells were washed twice with 1X PBS at 150 *μ*L/well. Then, 100 *μ*l of streptavidin-peroxidase (KPL) 1/1,000 diluted in PBS-BSA 1% was added per well, and the plates were incubated at room temperature. After 30 minutes, the cells were washed twice with 1 × 150 *μ*l PBS per well. Development was performed with the chromogen 3-amino-9-ethyl carbazole (AEC kit, Vector) according to the manufacturer's instructions, adding 100 *μ*l/well. The reaction was usually stopped with distilled water 20 minutes after adding the chromogen after removing the AEC. The cells were counted under an inverted light microscope by two experienced blinded observers to determine the number of FFUs, and the results obtained were averaged.

### 2.2. Treatment with Brefeldin A

To determine the role of inhibition of the cellular transport of viral NS1 in the efficiency of ZIKV FFA, brefeldin A (BD GolgiPlug cat: 555029) was added to a final concentration of 1 *μ*g/ml to part of the experiments. Taking into account the dynamics of NS1 secretion [8, 11, 12, 29, 30], BFA was added in three different schemes as follows: (i) after 1 hpi with a total infection time of 12 h (1 h–12 h), (ii) after 1 hpi with a total infection time of 24 h (1 h–24 h), and (iii) after 12 hpi with a total infection time of 24 h (12 h–24 h). During incubation in the presence of BFA, the cells were incubated at 37°C and 5% CO_2_. For each dilution, 6 replicates were performed.

### 2.3. Focus-forming Assay for ZIKV

To determine the detection efficiency of the combined FFA, spiked experiments were designed. A known quantity of 30,000 FFU of ZIKV-PR was added to plasma from patients seronegative for circulating IgG ZIKV and DENV NS1. This same viral quantity was added to the plasma of patients with a history of ZIKV infection confirmed by RT-qPCR who were seropositive for IgG ZIKV NS1 detected by ELISA and used as controls [31]. The plasma samples were incubated for 30 min at 57°C before application to cells. Semiconfluent Vero-76 cells were treated for 24 h with patient plasma in the presence or absence of ZIKV serially diluted in complete medium with 2% FBS, with a total volume of 100 *μ*l added per well. The cells were incubated at 37°C and 5% CO_2_. Each dilution was performed in duplicate. At the end of the ideal time, the combined FFA described above was performed using ZIKV NS1-specific mAbs.

### 2.4. Detection of Natural DENV Infection in Clinical Specimens from Febrile Patients

Serum/plasma samples from patients of emergency services at first-level hospitals in the Department of Huila 2019 who had primary acute DENV-1 or DENV-2 infection confirmed by conventional RT-PCR and the absence of IgG-DENV determined by an NS1-based ELISA were selected for the trial. Subsequently, the samples were serially diluted in complete medium with 2% FBS, starting with a 1/4 dilution, and a volume of 100 *μ*l was added per well. The samples were incubated for 24 h at 37°C and 5% CO_2._ At the end of the designated time, the described FFA for DENV was performed. Each dilution was performed in duplicate.

This study was approved by the Research Bioethics Committee of the Facultad de Salud de la Universidad Surcolombiana (approval code: 1745-2019).

### 2.5. Digital Image Analysis

To analyze the intensity of FFA immunostaining at the single-cell level, the open-source software CellProfiler™ was used [32]. Of note, this software is a free-access semi-automated image processor highly used in studies, particularly in immunofluorescence microscopy [27, 32, 33]. For the use, acquisition, and analysis of collected images, quality criteria and previously published protocols were used [34]. A Motic AE31-inverted microscope with an attached digital camera (Moticam BTU10, reference 17023473, 5 MP) was used to capture the images. Images of the FFU assays were taken using the same light intensity with the 10X objective. The image analysis path used in the software was as follows: Crop, ColorToGray, ImageMath, Identify PrimaryObjects, MeasureObjectIntensity, and ExportToDatabase. In the ColorToGray module, each image was divided into RGB channels. The green channel was used in the ImageMath module to invert the image and achieve a black background with white spots. For the detection of the focus-forming units (FFU) and their discrimination from the background (Background) by IdentifyPrimaryObjects, the size used ranged from 20 to 100 pixels in diameter. All methods available for the discrimination procedure were tested. Otsu was chosen from three classes due to assigning intermediate pixels as foreground, which was the best differentiation method in our case. The lower threshold limit was set based on the signal intensity of mock-treated Vero-76 cells. The intensity of each spotlight was measured in the MeasureObjectIntensity module. The database was exported to SQLite format and read in the open-source tool DB Browser, where the data of the average of the arbitrary intensity units (AIU) for each FFU were extracted for analysis. Usually, 200 FFUs per condition were included for analysis.

### 2.6. Statistical Analysis

The data were stored in a Microsoft Excel spreadsheet with a student Office 365 A1 Plus license. For statistical analysis, whether the data from each set of experiments had a normal distribution was initially determined using the Shapiro–Wilk test. The values were expressed as the mean and standard deviation or as the median (range) when the distribution was normal or non-normal, respectively. For nonparametric data, Mann–Whitney tests were performed to compare two independent variables, and the Kruskal–Wallis test and Dunn's posttest were used to compare more than two independent variables [35]. For parametric data, *t* tests were performed for two independent variables, and ANOVA and Tukey's test were performed for more than two variables. Analyses were carried out using GraphPad Prism version 8.0 software. *p* < 0.05 was considered significant in all cases.

## 3. Results

mAb 644 for ZIKV and mAb 323 for DENV had better efficiency than mAb 130 and mAb 271 in the FFAs.

A panel of mAbs that specifically detect the NS1 protein of ZIKV (mAb 644 and mAb 130) and DENV (mAb 323 and mAb 271) were initially tested in the FFA. Of note, the virus specificity of this group of murine mAbs was previously tested [28, 36].

**Figure 1:**
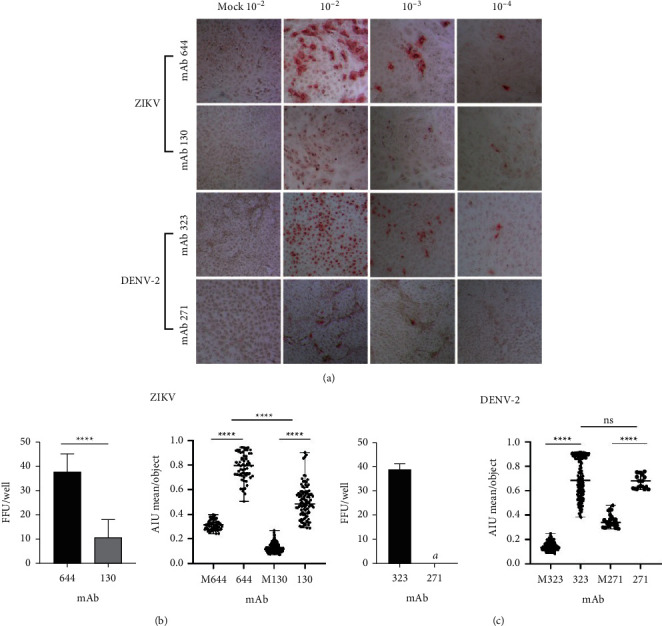
mAb 644 and mAb 323 were more efficient in identifying infectious viral particles of ZIKV and DENV-2, respectively. FFAs were performed at 24 hpi with all antibodies tested at a concentration of 1 *μ*g/ml. (a) Optical microscopy photographs of FFAs at 10x magnification, comparing different serial dilutions at base 10 in terms of the FFU detected with the different anti-NS1 ZIKV and DENV-2 antibodies and their respective Mocks. (b) Left: Mean ± standard deviation of the number of FFUs counted in the wells at a dilution of 10^−4^ for each of the mAb analyzed. Right: Median ± range of the average AIU per FFU measured for each antibody utilizing the CellProfiler™ free software. M323: mean Mock-treated cells stained with mAb 323. (c) Left: Mean ± standard deviation of FFUs counted in wells at the 10^−4^ dilution for each anti-DENV antibody. Right: Median ± range of the average AIU per FFU measured for each antibody using the CellProfiler™ free software. Significant differences between antibodies according to the unpaired *t* test or Mann–Whitney test are denoted by asterisks (*p* < 0.0001). *a*: No foci were found at this dilution. The experiments were carried out in replicates of 6 for each dilution.

The mAbs panel for both viruses was initially tested in the FFA at a concentration of 1 *μ*g/mL and a time of 24 hpi (Figure 1(a)). Of the ZIKV NS1 mAbs tested, mAb 644 was superior to mAb 130 in detecting infective viral particles, with a 3.5-fold higher absolute number of FFUs/well (Figure 1(b)) and a higher intensity of digitally assessed FFUs, with AIU ranges of 0.82 (0.51–0.93) and 0.4 (0.22–0.90) for mAb 644 and mAb 130, respectively (*p* < 0.0001, ANOVA test) (Figure 1(b)). The viral titers determined with the two ZIKV NS1-specific mAbs were 3.7 × 10^6^ FFU/mL for mAb 644 and 1.1 × 10^6^ FFU/mL for mAb 130. The greater intensity of the FFUs facilitated counting, in addition to the greater number of FFUs detected ensuring more precise quantification. Based on the above results, mAb 644 was selected for subsequent use in all ZIKV FFAs.

For the FFA of DENV-2, mAb 323 was superior to mAb 271 in detecting the absolute number of viral particles, with significant differences in the number of FFUs detected but not in the intensity calculated for each focus obtained (Figure 1(c)), reflecting a difference between the two methods of viral titer quantification. The results were 3.9 × 10^6^ FFUs/mL for mAb 323 and 6.8 × 10^3^ FFUs/mL for mAb 271, although the intensity in the foci did not present significant differences (*p*=0.71, Mann–Whitney test). Based on these results, in all remaining FFA experiments for DENV-2, mAb 323 was used.

### 3.1. Identification of the Optimal Concentration of mAbs Used in the FFAs

**Figure 2:**
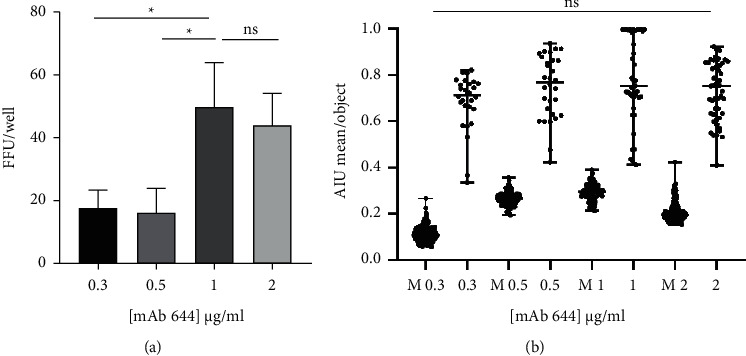
Selection of the optimal concentration of the mAb for carrying out the ZIKV FFA. The FFA was performed at 24 hpi, and different concentrations of mAb 644 anti-NS1 ZIKV were used as the primary antibody. (a) Mean ± standard deviation of the number of FFUs/well counted at the 10^−3^ dilution for each antibody concentration.^*∗*^*p* < 0.05, Tukey's posttest for multiple comparisons. (b) Median ± range of the average AIU per FFU measured for each antibody concentration with the CellProfiler™ free software. ^*∗*^*p* < 0.05, Dunn's posttest. M: Mock.

To obtain the optimal concentration of each mAb for use in immunocytostaining, FFAs at 24 hpi were performed to test concentrations of 0.3, 0.5, 1, and 2 *μ*g/mL of mAb 644 as the primary antibody for ZIKV FFU detection.  Figure 2(a) shows a significantly higher number of FFUs obtained at a concentration of 1 *μ*g/mL compared with the lower concentrations of 0.3 and 0.5 *μ*g/mL (*p*=0.016 and *p*=0.013, Dunn's posttest, respectively). No difference in the number of FFUs/well was found when concentrations of 1 and 2 *μ*g/mL of mAb 644 were used as the primary antibody. When the intensity of each focus was digitally analyzed (Figure 2(b)), no significant differences in foci intensity were found among any of the 4 concentrations tested. Thus, based on the above results, the 1 *μ*g/mL concentration of mAb 644 was selected for subsequent performance of all FFAs. This ideal concentration was also used for mAb 323 to detect DENV-2 NS1, based in previous results and the similar characteristics such as the isotype and performance in the ELISA [28, 36].

### 3.2. Selection of the Optimal Infection Time for the FFAs

Considering the rapid dynamics of *flavivirus* NS1 expression in susceptible cells [12, 13, 37, 38], we then determined the optimal infection time that offered the best efficiency in ZIKV FFU detection. For this, ZIKV infection of Vero-76 cells was performed in 96-well microplates, and at 6, 12, 24, and 48 hpi, FFAs were performed to finally evaluate the FFUs/well detected in the 10^−4^ dilution at each of the times tested. The count in this dilution was selected due to the ability to distinguish and count the foci at most of the analyzed postinfection times.

**Figure 3:**
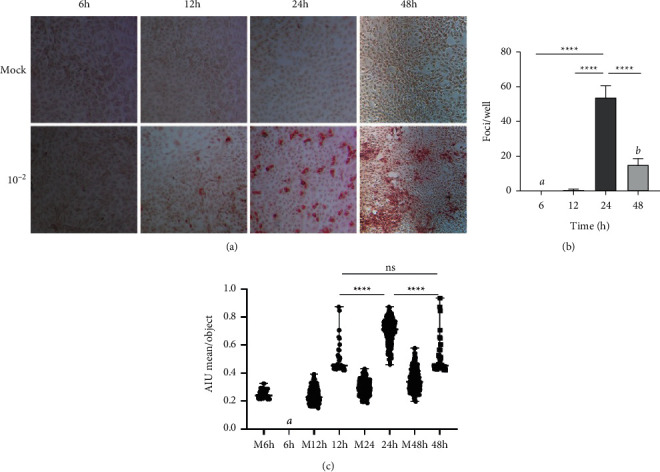
Twenty-four hours was the optimal time for performing the ZIKV focus-forming assay. (a) Comparison of the FFUs at different times in the 10^−2^ dilution. (b) Number of FFUs/well counted at the 10^−4^ dilution at each time postinfection. The mean ± standard deviation is shown. ^*∗∗∗∗*^*p* < 0.0001, Tukey's test. (c) Arbitrary intensity unit (AIU) values found in the FFUs at each postinfection time, as evaluated by the free software CellProfiler^TM^. The median and range are displayed. ^*∗∗∗∗*^*p* < 0.0001, Dunn's test. *n* = 2 experiments in triplicate. *a* Not detectable. *b* Unreliable foci count due to the formation of clusters of infected cells. M: Mock.

As shown in Figure 3(a), at 6 hpi, no foci were detected in the cultures. When the 12 hpi were evaluated, a low but clear number of FFUs was observed in the analyzed dilution (Figure 3(a)). A significantly higher number of FFUs/well were observed at 24 hpi, with a mean (±SD) of 53.7 ± 6.9 FFUs/well, compared with the two previously mentioned times (Figure 3(b)), with a *p* < 0.0001 according to Tukey's posttest for both comparisons (Figure 3(b)). When the intensity of each focus was evaluated in a semi-automated way, higher AIU values were found at the 24 hpi median (range), measuring 0.71 (0.46–0.87), compared to all other times tested, with *p* < 0.0001 according to Dunn's posttest (Figure 3(c)). Finally, titers of 5.5 × 10^4^ at 12 hpi, 5.3 × 10^6^ at 24 hpi, and 48 hpi of 1.1 × 10^6^ FFU/mL were obtained (data not shown). However, as noted in Figure 3(a), at 48 hpi, the foci showed a tendency to converge, forming a large number of clusters of infected cells in an effect possibly explained by viral replication and dispersion to neighboring cells presented at this time, thus causing a loss of FFU definition and making an accurate count more difficult. Based on the above findings, the postinfection time selected to perform the FFAs was 24 h.

### 3.3. Effect of Brefeldin-A on the Efficiency of FFA for ZIKV

**Figure 4:**
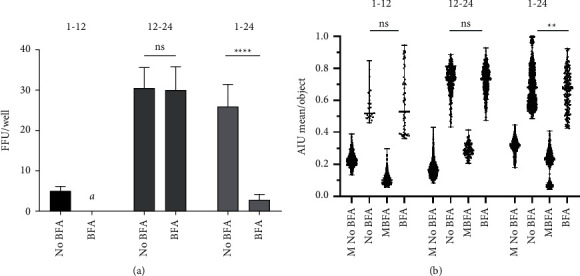
BFA did not improve the focus-forming assay. The three schemes shown at the top of Figure 4(a) and 4(b) were tested in this set of experiments. The first number represents the incubation time without BFA, and the second number is the time when the FFA was revealed, as shown in hpi. (a) Number of FFUs counted in the six wells at the 10^−4^ dilution for each scheme shown. The mean ± standard deviation is presented. ^*∗∗∗∗*^*p* < 0.0001, Mann–Whitney test. (b) Median ± range of the average arbitrary intensity units per FFU evaluated under each condition with the CellProfiler™ free software. ^*∗∗*^*p* < 0.01, Mann–Whitney test. M: Mock. *n* = 2 experiments with serial dilutions with 6 replicates for each dilution.

To assess whether increasing the accumulation of intracellular NS1 by inhibiting protein transport in turn increases the efficiency of FFU for ZIKV, the trans-Golgi inhibitor brefeldin-A was added to the cultures at a final concentration of 1 *μ*g/ml, as previously described [13]. As a control, Vero-76 cells to which a vol/vol ratio of sterile 1X PBS was applied, which was similar to the volume of brefeldin-A applied to the tested cultures, were used (no BFA condition). For this experiment, Vero-76 cells were infected with ZIKV and maintained in 3 different incubation schemes: (i) 1 hpi without BFA, followed by 12 h in the presence of BFA (1 h–12 h); (ii) 1 hpi with a following 24 h of infection in the presence of BFA (1 h -24 h); and (iii) 12 hpi without BFA with the final hours in the presence of BFA, for a total of 24 h of infection (12 h -24 h). As shown in Figure 4(a) and consistent with the previous results shown here, the number of FFUs found in cells without BFA at 12 hpi was significantly lower than that found at 24 hpi. When the FFU number was analyzed in Vero-76 cells infected with ZIKV for 24 h with the last 12 h in the presence of BFA, no difference was found relative to cells not treated with BFA (Figure 4(a)). ZIKV-infected Vero-76 cells that had been in prolonged contact with BFA (schemes 1–24) showed a significant drop in the number of FFUs/well found, which was an effect associated with drug-induced cell toxicity (Figure 4(a)) observed as in the loss of monolayer integrity in both mock-and BFA-treated infected cells (data not shown).

Automated analysis of digital images of the cell density showed no differences between FFU treated and untreated with BFA under conditions (1 h -12 h) and (12 h -24 h), also ruling out relative differences in intracellular NS1 accumulation induced by BFA (Figure 4(b)). However, long-term exposure to BFA (schemes 1–24) showed a significant drop in AIU compared with cells not treated with BFA and cultured for the same time period, which was an effect possibly due to the toxicity of BFA in such a treatment scheme (Figure 4(b)). In summary, the application of a trans-Golgi inhibitor during the FFU assay for ZIKV did not improve the number and density of FFUs found, and after 24 h of exposure, BFA had a significant cytotoxic effect on Vero-76 cells.

### 3.4. Efficiency of FFA Detection of ZIKV in Plasma

To determine the efficiency of the FFA in the detection of infective viral particles in plasma, where it is known that multiple proteins and lipid factors can interfere with the efficiency of the assay [38], a group of ZIKV spike recovery experiments were performed.

**Figure 5:**
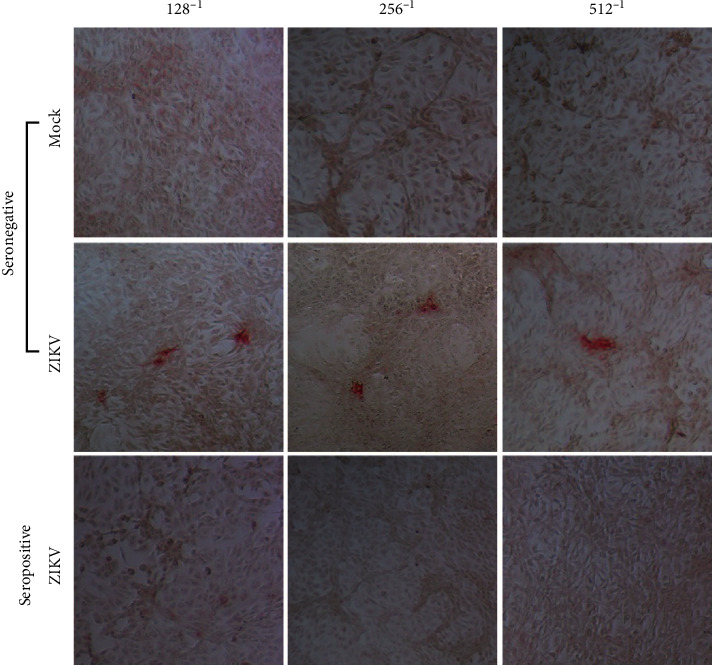
FFAs of plasma from patients after contacting ZIKV-PR *in vitro.* Conventional light microscopy photographs at 10X. Mock or ZIKV was added in known amounts to plasma specimens from ZIKV-IgG-seronegative or ZIKV-IgG-seropositive patients and incubated for 1 h at room temperature. The FFAs were performed under optimal conditions. The foci detected at the respective dilutions of the plasma incubated with ZIKV-PRVA BC59 are shown. Seronegative (patient code SU 673) and seropositive (patient code ZM 163). A representative experiment of three replicates is shown.

For this evaluation, known amounts of ZIKV FFU were added to plasma from patients seronegative and seropositive for specific ZIKV-NS1 IgG, and subsequently, FFAs were performed. The virologic and serological characteristics of the included patients are shown in Table 1.  Figure 5 shows a representative experiment of 3 assays performed in triplicate. As expected, no FFUs were observed when Vero-76 cells were treated with the mock solution (Figure 5). On the other hand, a median (range) of 1.19 × 10^5^ (1.18 × 10^5^–1.19 × 10^5^) FFU/mL was obtained in spike assays performed with plasma from patients seronegative for ZIKV IgG. Demonstrating the specificity of the assay, this response was inhibited in experiments where ZIKV was added to the plasma of IgG-seropositive patients with confirmed ZIKV infection at all dilutions tested (1/2 to 1/1,024), demonstrating the effect of circulating neutralizing antibodies that inhibited the entry of the added ZIKV into Vero-76 cells (Figure 5).

As shown in Table 2, >98% recovery of infecting ZIKV viral particles was obtained with the ZIKV FFA.

### 3.5. Detection of DENV-1 and DENV-2 Viral Particles in Clinical Specimens from Febrile Patients with Natural Infection

The results presented here demonstrated the utility of the developed FFA for the detection of ZIKV produced in cell culture. Subsequently, we wanted to determine the usefulness of our FFA in the detection of circulating DENV in plasma from patients with natural infections. For this evaluation, plasma from 3 febrile patients with primary infection (based on the absence of IgG-DENV in the acute phase) by DENV-1 and DENV-2 was selected (Table 1). The reason for evaluating patients with primary infection was to eliminate the possibility of previously existing anti-DENV antibodies in circulation that could interfere with the detection of circulating infecting viruses in the FFA. The characteristics of the patients evaluated are shown in Table 1.

**Figure 6:**
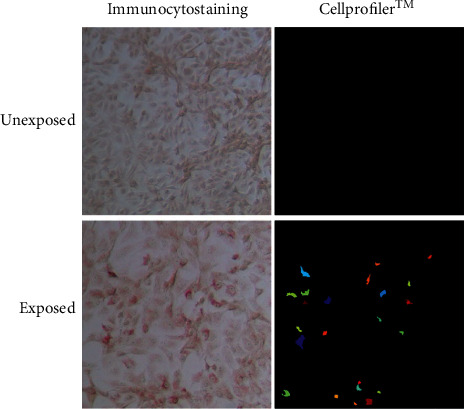
FFAs of natural DENV infection. 10x magnification factor. The plasma was diluted 4 times. The different foci detected automatically by the free software CellProfiler™ are marked by color, and the threshold used to detect the foci was determined based on the unexposed foci. Representative images of three sera tested in duplicate are shown.

As shown in Figure 6, clear foci of infecting DENV-2 viral particles were detected at a dilution of 1/4 with the FFA developed here. The optical microscopy images obtained with the FFA of natural DENV infection were 98% concordant with the number of FFUs identified by the automated digital image analyzer (Figure 6), allowing easy and precise quantification of the viral titers present in plasma samples from patients with acute primary infection. The summary of the results of the 3 experiments carried out is shown in Table 3, displaying the obtained titers of infectious viral particles of DENV in natural infections. To our knowledge, this is the first report of the detection of natural DENV infection by an FFA combined to automated analysis of digital imaging. The results presented here are comparable to those rarely reported in the detection of natural infection by plaque-forming assay (PFA) [40].

## 4. Discussion

In this study, we optimized an FFA to quantify infectious particles of ZIKV and DENV in culture supernatants and clinical specimens using the combination of conventional light microscopy with automated digital image analysis and found the following: (1) the best mAbs and their ideal concentrations for use in the FFAs were identified, (2) the infection time of 24 hpi was found to be optimal, (3) the inhibition of protein transport through the Golgi apparatus did not modify the efficiency of the FFA, and (4) these assays could be used to detect ZIKV and DENV in cell culture supernatants and naturally infected clinical specimens, respectively.

The purpose of the FFA is to detect infectious viral particle. In this assay, intracellular NS1 was detected, unlike other assays that detect structural proteins [12, 19, 21, 41]. Using the detection of NS1 as the basis of the FFA offers the advantage of evaluating a protein that is not part of the infectious viral particle and that is expressed only during active viral replication, avoiding the risk of evaluating the viral input of the infection present in the inoculum.

In this assay, a novel combinational technique of conventional enumeration and automated digital image analysis was also used for *flavivirus*. The advent of this technology has generated great advances in this field, even though these types of tools are more widely used in assays of fluorescence. In the assay developed here, the use of digital image analysis served as a decision-making means for the selection of appropriate conditions, such as the mAbs, concentration, and ideal infection times, providing precision and reducing the subjectivity of manual optical enumeration and counting. Most studies that use digital analysis combined with immunocytostaining do so to increase the counting accuracy [26, 27]. In our assay, in addition to the above reason, this digital tool was used to evaluate additional parameters such as the intracellular intensity produced by staining with the antibody system. The relative intracellular intensity quantification indirectly represents the relative amount of the target intracellular protein.

As shown in Figure 1, mAb 644 was superior to mAb 130 for the identification of ZIKV NS1 in both the absolute number and intensity obtained digitally. According to previous reports [28], mAb 130 specifically identifies NS1 of ZIKV, with low cross-reactivity for the NS1 of DENV, and this antibody has been used for the development of immunochromatographic and enzyme-linked immunoassay tests for the detection of ZIKV [28]. mAb 644, which is highly specific for ZIKV-NS1, also showed the best performance in this study. mAbs 323 and 271 have been widely characterized [36], and both have been used for the detection of the four DENV serotypes NS1 in immunochromatographic and ELISAs. In our case, mAb 323 showed better efficiency in detecting foci in DENV infection (Figure 1). Both mAbs recognize an epitope on “wing” domain of the NS1 DENV, and both have been considered pan-DENV as they bind to NS1 from the 4 serotypes. However, mAb 271 has shown higher affinity to individual serotypes such as DENV-3 and DENV-1 [28, 36]. Its differences could explain the better results obtained with mAb 323 in the FFA.

The concentration of the chosen antibodies that performed best in the FFAs was 1 *μ*g/ml (Figure 2). The working concentrations of the primary antibodies used by other researchers in FFAs vary from 0.3 to 1 *μ*g/ml [30, 42, 43], although most antibodies used are directed against viral structural proteins, such as *E* or preM. Antibodies that recognize these structural proteins generally have significant cross-reactivity between flaviviruses [44, 45].

There is variability in the previously reported incubation times required for these techniques. In studies carried out for ZIKV, incubation times from 30 h to 72 h were utilized [9, 36]. For DENV ranging from 48 h to 72 h [26, 29], WNV ranged from 18 to 24 h [29, 30]. We showed that the optimal incubation time was 24 h. For both viruses, shorter times did not produce detectable foci, and with longer times, the overlapping of foci was observed (Figure 3(a)). The use of NS1 as the target viral protein for detection in FFAs could explain the short incubation time for the selected FFAs. NS1 is a nonstructural protein with rapid expression in infected cells. After viral entry *in vitro*, the secretion of NS1 has been described to occur within times as short as 12 h [13].

For cell lines derived from mammalian cells, such as Vero-76 (kidney epithelial cells derived from African green monkey), it has been shown that DENV NS1 traffics through the classical pathway [12, 13]. Therefore, we used BFA at 1 *μ*g/ml to theoretically increase the intracellular concentration of NS1 by blocking trans-Golgi trafficking and attempted to improve the assay overall. However, the application of this drug to cultures with different FFA incubation times did not induce an improvement in the detection of the absolute number of FFUs or the intracellular intensity analyzed at the single-cell level (Figure 4). This could be explained by various factors. First, BFA has been reported to be an inhibitor of viral replication in the early stages of the viral cycle [46, 47], which is consistent with the observations found here, where a decrease in the viral titer was observed only in cells treated with BFA after 1 h of infection (Figure 4(a)). On the other hand, NS1 secretion does not occur exclusively by the classical pathway, as reported by Alcalá et al., and treatment with BFA decreased NS1 secretion by only 35% [13]. In addition, the FFA detects NS1 anchored to the membranes of the endoplasmic reticulum or the Golgi. Soluble proteins are not adequately bound by the methanol used for binding in the assay; thus, it is possible that the inhibition of the classical protein transport pathway did not increase NS1 protein adherence to intracellular membranes.

The detection of circulating DENV and ZIKV in natural infection using FFAs and plaque-forming assays (PFA) is hardly possible [40]. FFAs reported for *flavivirus* infections are generally used in *in vitro* experiments or to determine the detection limits of molecular tests such as RT-PCR [20], as well as to measure neutralization potentials in various studies [24, 48]. We demonstrated that the optimized assay detects circulating viral particles in natural DENV-1 and DENV-2 infections (Figure 6 and Table 3), which is particularly useful under conditions wherein several DENV serotypes cocirculate. The inhibition of ZIKV FFU detection by plasma from ZIKV-IgG-seropositive patients demonstrated that the assay presented here has the potential to detect relative amounts of neutralizing antibodies (Figure 5 and Table 2), key humoral immune factors in vaccine evaluations, and new antiviral drugs [49–51]. This study has some limitations. The FFA need be extended to other serotypes such as DENV-3 and DENV-4 and a bigger group of experiments focused to determine the neutralizing antibody activities against DENV and ZIKV should be keep in mind.

In conclusion, the combination of the two techniques optimized the FFAs, allowing the study of DENV and ZIKV in culture supernatants and clinical specimens from natural infection in hyperendemic areas where the two viruses cocirculate.

## Figures and Tables

**Table 1:** Virological and serological characterization of the clinical samples used.

Code	RT-PCR	NS1-DENV	IgM-DENV	IgG-DENV	IgG-ZIKV
SSH 6	DENV-1	+	−	−	NR
SSH 7	DENV-1	+	−	−	NR
SSH 12	DENV-2	+	−	−	NR
SSH 23	−	−	−	−	NR
SSH 15	−	−	−	−	NR
SU 673	−	−	−	−	NR
ZM 163^a^	ZIKV	NR	NR	NR	+

a. Patient with confirmed ZIKV infection by RT-qPCR in the sample prior to be used. NR: not realized.

**Table 2:** Spike recovery assay results for ZIKV in FFA.

	ZIKV particles			
	Dilution	Expected	Obtained	% Recovery
ZIKV-NS1 IgG seronegative	128^−1^	30,000	29,568	98,56
	256^−1^	30,000	29,184	97,28
	512^−1^	30,000	28,672	95,57

ZIKV-NS1 IgG seropositive	128^−1^	30.000	0	0
	256^−1^	30.000	0	0
	512^−1^	30.000	0	0

**Table 3:** Viral titers found in plasma samples from naturally infected patients.

Shows	FFU/mL
SSH 6	2.58 × 10^3^
SSH 7	2.00 × 10^3^
SSH 12	6.50 × 10^2^
SSH 23	ND
SSH 15	ND
SU 673	ND

ND. Not detectable.

## Data Availability

Data used to support the findings of this study are available from the corresponding author upon request.
